# Supplementary material for: Phage family classification under Caudoviricetes: A review of current tools using the latest ICTV classification framework
Source: Front Microbiol. 2022 Dec 16;13:1032186. doi: 10.3389/fmicb.2022.1032186 (PMC9800612; doi:10.3389/fmicb.2022.1032186)
Supplement: Supplementary file 1 [file Data_Sheet_1.PDF]

frontiers

Phage family classification under Caudoviricetes: a review of current tools using the latest ICTV classification framework

1 DATA AVAILABILITY

1.1 RefSeq dataset

Data source: `https://www.ncbi.nlm.nih.gov/labs/virus/`

The query used for retrieval from the NCBI Virus database included the following: Virus [Caudoviricetes, taxid:2731619] — Sequence Type [RefSeq] — Refseq Genome Completeness [complete]

1.2 Simulated metagenomic dataset:

The paper of data source: `https://arxiv.org/abs/2201.04778`

The labeled data can be downloaded from: `https://github.com/JudithEllyn/Simulated`

2 TOOL USAGE INSTRUCTION

2.1 MMseqs2

Source of the code: `https://github.com/soedinglab/MMseqs2`

Reference code: `https://github.com/apcamargo/ictv-mmseqs2-protein-database`

Command:

- mmseqs createdb –dbtype 1 {DB.fasta} {targetDB}
- mmseqs createtaxdb {targetDB} tmp –ncbi-tax-dump {taxonomy folder} –tax-mapping-file {fna.taxidmapping}
- mmseqs easy-taxonomy {QUERY.fasta} {targetDB} {alnResult} tmp -e 1e-5 -s 6 –blacklist “” –tax-lineage 1

2.2 PhaGCN

Source of the code: `https://github.com/KennthShang/PhaGCN_newICTV`

Command: python run_Speed_up.py –contigs {QUERY.fasta} –len 3000

2.3 CAT

Source of the code: `http://github.com/dutilh/CAT`

Command:

- CAT prepare –db_fasta {DB.fasta} –names {names.dmp} –nodes {nodes.dmp} –acc2tax {acc2taxid.txt.gz} –db_dir {database folder}

- CAT contigs -c {QUERY.fasta} -d {database folder} -t {taxonomy folder} –out_prefix {output prefix}
- CAT add_names -i {ORF2LCA / classification file} -o {output file} -t {taxonomy folder} –only_official

2.4 vConTACT 2.0

Source of the code: `http://bitbucket.org/MAVERICLab/vcontact2/src/master/`

Command:

- python bin/vcontact2_gene2genome -p {QUERY.faa} -o {gene-to-genome mapping file} -s 'Prodigal-FAA'
- vcontact2 –raw-proteins {QUERY.faa} –rel-mode Diamond –proteins-fp {gene-to-genome mapping file} –db {DB version} –pcs-mode MCL –vcs-mode ClusterONE –c1-bin {path/to/clusterone} –output-dir {target output folder}
